# From Sensory Deprivation to Psychosis: Environmental Mechanisms of Delirium in Dementia—An Eight-Year Retrospective Longitudinal Case Report and Narrative Review

**DOI:** 10.3390/healthcare14172705

**Published:** 2026-08-25

**Authors:** Paulina Teodorczyk, Anna Różańska-Walędziak

**Affiliations:** Human Physiology and Pathophysiology Department, Faculty of Medicine, Collegium Medicum, Cardinal Stefan Wyszynski University in Warsaw, 01-938 Warsaw, Poland

**Keywords:** delirium superimposed on dementia (DSD), sensory deprivation, visual impairment, psychotic symptoms, non-pharmacological interventions, environmental instability

## Abstract

**Background/Objectives**: Delirium in patients with dementia is a multifactorial condition traditionally attributed to acute medical triggers. However, emerging evidence suggests that non-biological factors, including sensory deprivation and environmental instability, may contribute to delirium vulnerability, particularly in vulnerable populations. To explore the temporal associations between non-biological and environmental factors, particularly sensory deprivation and recurrent delirium in a patient with advanced Alzheimer’s disease and progressive vision loss, a narrative review was conducted. **Methods**: A narrative review of the literature on delirium risk factors, differential diagnosis, and non-pharmacological interventions was conducted. A retrospective longitudinal case report of a patient with advanced Alzheimer’s disease and progressive visual impairment, based on an eight-year observation, was used to contextualize the literature findings. **Results**: The literature indicates that delirium arises from the interaction between predisposing factors, such as cognitive impairment and reduced cognitive reserve, and precipitating factors, including both biological and environmental stressors. Sensory deprivation, particularly visual impairment, may significantly disrupt orientation and increase reliance on internally generated perceptual content, predisposing to hallucinations and delusions. Environmental instability, such as changes in caregivers, disrupted routines, and overstimulation, may further exacerbate this vulnerability. Evidence suggests that these factors may contribute to delirium even in the absence of identifiable classical medical triggers. In the presented case, individualized non-pharmacological interventions based on structured routines, environmental predictability, and sensory-adapted engagement were associated with periods of improved behavioral stability. **Conclusions**: The present case supports the hypothesis that delirium in dementia may involve impaired environmental integration, particularly in the context of sensory deprivation, in addition to established biological mechanisms. Person-centered non-pharmacological strategies aimed at maintaining familiarity, environmental predictability, and appropriate sensory support may represent valuable components of delirium prevention and management. These hypothesis-generating observations require confirmation in prospective studies involving larger patient populations.

## 1. Introduction

Delirium superimposed on dementia (DSD) is one of the most common neuropsychiatric syndromes affecting older adults with cognitive impairment and represents a major clinical challenge. Compared with individuals without dementia, patients with pre-existing cognitive decline are substantially more susceptible to delirium due to reduced cognitive reserve and increased vulnerability to physiological and environmental stressors [1,2]. The occurrence of DSD is associated with prolonged hospitalization, accelerated functional and cognitive decline, institutionalization, and increased mortality, making its prevention and early recognition a priority in geriatric care [3].

Although delirium is traditionally characterized by disturbances in attention, awareness, and cognition, psychotic manifestations are also common, particularly in older adults with dementia. Visual hallucinations, delusions, perceptual distortions, and severe misinterpretations of environmental stimuli frequently accompany delirium episodes and are associated with greater symptom severity, increased patient distress, and a higher burden on caregivers. These manifestations often complicate the differential diagnosis with primary psychotic disorders or dementia with Lewy bodies, especially in patients with advanced neurodegenerative disease. Consequently, understanding the mechanisms underlying psychotic symptoms in delirium remains an important clinical and research priority [4,5].

Because delirium is potentially reversible, current diagnostic algorithms prioritize the exclusion of acute medical precipitants such as infection, metabolic abnormalities, dehydration, and medication-related adverse effects. Nevertheless, these factors do not fully explain recurrent delirium in all patients with dementia [6].

Visual impairment is a well-established predisposing factor for delirium and is consistently associated with an increased risk of adverse neuropsychiatric outcomes in older adults. Severe visual loss limits access to environmental cues that support orientation, reduces the accuracy of sensory processing, and increases reliance on internally generated perceptual representations when external sensory information is insufficient [7,8]. Consequently, patients with dementia and profound visual impairment may become particularly vulnerable to visual hallucinations, delusions, and misinterpretation of their surroundings [9]. However, despite growing recognition of sensory impairment as a risk factor for delirium, little is known about how progressive sensory deprivation interacts with environmental instability to trigger recurrent psychotic symptoms in patients with advanced dementia.

Although pharmacological treatment may be necessary to control severe agitation or psychotic symptoms that threaten patient safety, current clinical guidelines emphasize that medications should not be considered the primary treatment for delirium. Instead, pharmacotherapy should be reserved for carefully selected patients after reversible causes have been identified and addressed. The limited efficacy of pharmacological interventions in preventing recurrent delirium episodes highlights the need to better understand non-biological mechanisms contributing to symptom development [6,10].

Beyond biological precipitants, increasing attention has been directed toward the role of the care environment in maintaining orientation and emotional security in people with dementia. Familiar surroundings, consistent daily routines, continuity of caregivers, and the reduction in unnecessary environmental stressors are increasingly recognized as important components of person-centered care [11]. Environmental predictability and continuity of care help preserve orientation and reinforce a sense of security, particularly in patients with anxiety and advanced dementia. Such environmental stability may be particularly important in patients experiencing anxiety, as uncertainty and disrupted orientation can amplify fear, perceptual disturbances, and behavioral symptoms [12]. Nevertheless, the contribution of environmental consistency to the prevention of recurrent psychotic manifestations of delirium in patients with advanced dementia and severe sensory deprivation remains insufficiently explored.

Current evidence identifies sensory impairment as an important risk factor for delirium; however, its interaction with environmental factors has received considerably less attention. In particular, there is limited evidence explaining how progressive visual deprivation, anxiety, and disruption of familiar environmental cues contribute to recurrent psychotic symptoms in patients with advanced dementia in the absence of identifiable medical precipitants.

The particular contribution of the present report lies in the extended eight-year observation of a single patient with advanced Alzheimer’s disease in whom progressive visual impairment, recurrent delirium, and changes in environmental continuity could be examined across successive stages of clinical deterioration. Rather than focusing on a single acute delirium episode, the case provides an opportunity to describe temporal patterns between progressive sensory deprivation, environmental circumstances, and recurrent neuropsychiatric symptoms within the same patient. By integrating these longitudinal clinical observations with a narrative review of the literature, the report aims to generate hypotheses regarding the potential role of sensory and environmental vulnerability in recurrent delirium superimposed on dementia.

Therefore, this study aimed to explore the contribution of progressive visual deprivation and disruption of environmental continuity to recurrent psychotic manifestations of delirium in a patient with advanced Alzheimer’s disease through a longitudinal case presentation supported by a narrative review of the current literature.

## 2. Materials and Methods

### 2.1. Study Design and Rationale

This study was designed as a retrospective longitudinal case report supported by a narrative review of the literature. The case report describes the clinical course of a 94-year-old woman with Alzheimer’s disease who remained under continuous nursing and geriatric care over an eight-year observation period, from February 2018 to March 2026. The extended retrospective observation period allowed the evaluation of changes in cognitive and functional status, the progression of visual impairment, and temporal associations between environmental factors and recurrent episodes of delirium. Given the single-patient and retrospective nature of the study, these observations were considered hypothesis-generating and were not used to infer causal relationships.

### 2.2. Patient and Clinical Setting

The case involved a 94-year-old woman with a diagnosis of Alzheimer’s disease who had resided in a religious congregation for approximately 60 years and remained under continuous nursing and geriatric care throughout the observation period. The observation covered February 2018 to March 2026. During this period, the patient experienced progressive cognitive and functional decline accompanied by progressive visual impairment and recurrent episodes of delirium. Detailed clinical characteristics and the longitudinal course are presented in Section 3.

### 2.3. Data Sources and Procedure

The case was reconstructed through a retrospective review of nursing and geriatric medical records collected during routine clinical care. Particular attention was paid to the continuity of care throughout the observation period, allowing the identification of longitudinal changes in the patient’s clinical condition rather than the analysis of a single delirium episode.

The analysis included:

- Cognitive decline;
- Functional deterioration;
- Progression of visual impairment;
- Frequency and characteristics of delirium episodes;
- Occurrence of psychotic symptoms;
- Environmental circumstances associated with symptom exacerbation;
- Changes in pharmacological management.

A structured review of the patient’s pharmacotherapy was performed to evaluate the potential contribution of prescribed medications to delirium and behavioral and psychological symptoms of dementia (BPSD). Each medication was assessed with regard to its therapeutic indication, potential delirium risk, anticholinergic burden, and reported effects on neuropsychiatric symptoms based on current evidence.

The retrospective review of nursing and geriatric medical records was performed by the first author.

### 2.4. Clinical Assessment Instruments

Longitudinal clinical evaluation was based on routinely performed comprehensive geriatric assessments. Cognitive function was assessed using the Mini-Mental State Examination (MMSE), one of the most widely used brief screening instruments for the assessment of global cognitive impairment in clinical and research settings [13]. Because of progressive visual loss, the MMSE was modified during the later stages of follow-up by excluding visually dependent tasks, resulting in a maximum score of 26 points. Scores obtained using the modified 26-point version were interpreted as descriptive indicators of within-patient cognitive change and were not considered directly equivalent to scores obtained using the standard 30-point MMSE [14].

Functional independence was evaluated using the Katz Activities of Daily Living (ADL) Index, a widely established measure of basic functional ability with demonstrated reliability and validity in older adults [15,16,17].

Delirium was evaluated longitudinally throughout the eight-year observation period using the Confusion Assessment Method (CAM). The CAM is a validated diagnostic algorithm based on four core features of delirium—acute onset and fluctuating course, inattention, disorganized thinking, and altered level of consciousness—and has demonstrated high diagnostic accuracy in older adults [18]. All prospective CAM assessments were performed by the first author, who provided direct nursing care to the patient throughout the observation period.

During periods of clinically evident or suspected delirium, CAM assessments were conducted daily until symptom resolution. Outside of acute episodes, CAM evaluations and clinical observations were supplemented by a comprehensive review of routine nursing and geriatric documentation to capture the full frequency and characteristics of delirium episodes that may have occurred outside of active, predefined testing intervals. While prospective CAM evaluations guided acute clinical management, the retrospective integration of routine institutional records was necessary to reconstruct the longitudinal timeline of all reported episodes across the eight-year span.

### 2.5. Environmental Assessment

Environmental factors were analyzed using longitudinal nursing documentation and clinical observations recorded throughout the follow-up period. Particular attention was given to factors affecting environmental continuity, including changes in caregivers, disruption of daily routines, family visits, holidays, reduced activity, and other contextual events potentially associated with symptom exacerbation.

Environmental circumstances were identified from contemporaneous nursing documentation and clinical observations and were considered temporally associated with symptom exacerbation when they were documented in close temporal proximity to the onset or worsening of delirium symptoms. These associations were evaluated descriptively and were not interpreted as confirmed causal triggers.

### 2.6. Data Analysis

Given the single-patient retrospective case-report design, no inferential statistical analyses were performed. Clinical data were analyzed descriptively and chronologically to reconstruct changes in cognitive and functional status, visual impairment, delirium occurrence, psychotic symptoms, and relevant environmental circumstances over the eight-year observation period. Temporal co-occurrence between environmental changes and symptom exacerbations was examined descriptively and was not interpreted as evidence of causality.

**Figure 1:**
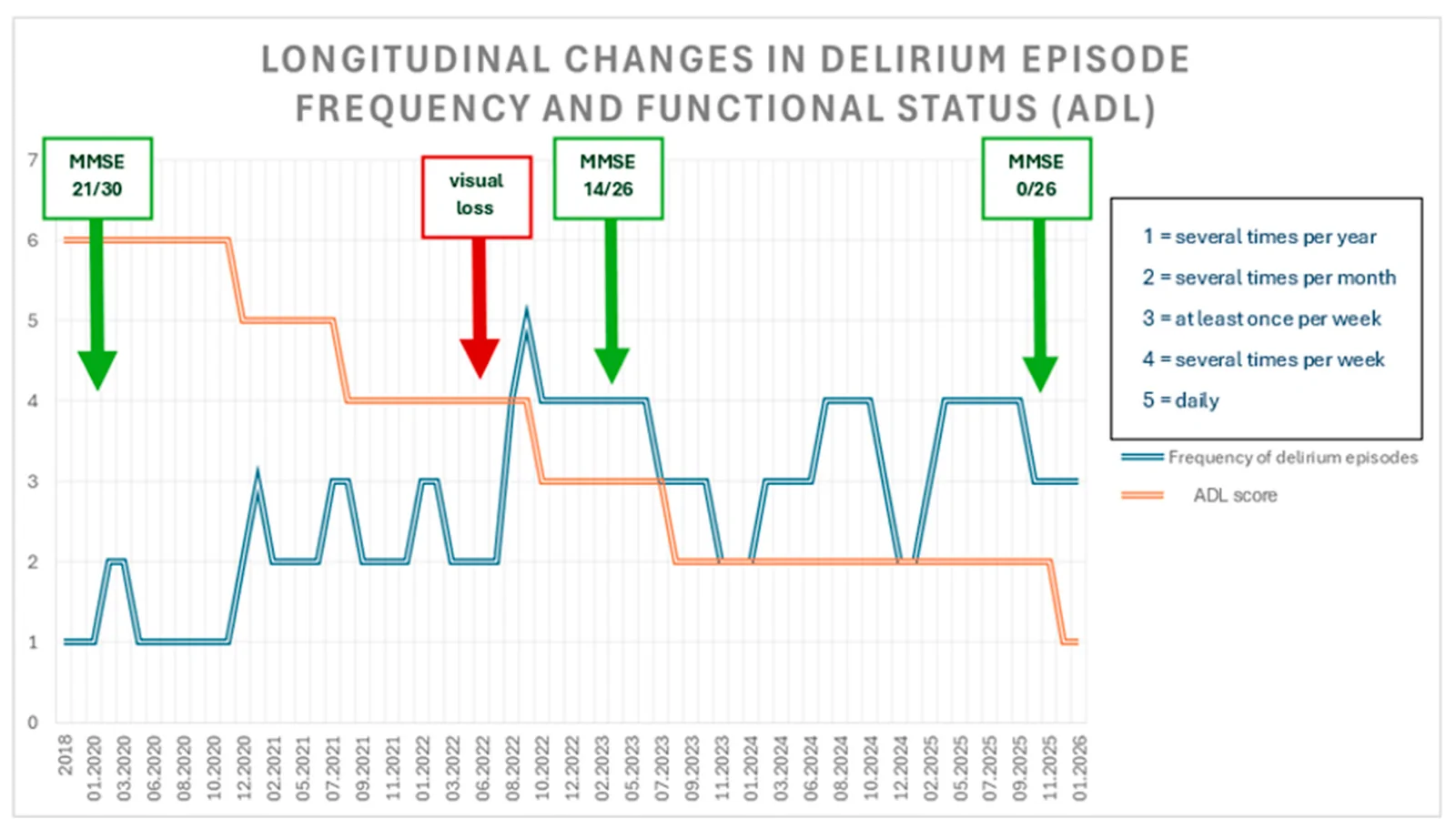
Longitudinal changes in delirium episode frequency and functional status (ADL) from 2018 to 2026. The frequency of delirium episodes shown in this figure represents a retrospective semi-quantitative synthesis based on prospectively documented CAM assessments and contemporaneous nursing documentation. The figure is intended to illustrate longitudinal clinical trends rather than exact quantitative measurements (1 = several times per year; 5 = daily). Functional status was evaluated using ADL score (range 0–6). MMSE scores are shown at key time points; due to progressive visual impairment, a modified MMSE version with a maximum score of 26 was used in later assessments.

To better illustrate the clinical course (Figure 1), the frequency of delirium episodes was presented using a semi-quantitative ordinal scale developed retrospectively on the basis of these prospectively collected CAM assessments and contemporaneous clinical documentation maintained throughout the entire observation period. The ordinal categories presented in Figure 1 were assigned retrospectively by the first author to reflect the relative frequency and clinical burden of delirium episodes during successive phases of the patient’s clinical trends [1].

The scale was not based on predefined threshold values or validated quantitative criteria. Its purpose was not to determine the absolute number of delirium episodes but to illustrate changes in their relative frequency and clinical burden over time. Accordingly, the classification is descriptive in nature and represents a retrospective clinical synthesis intended to visualize longitudinal clinical trends rather than exact quantitative measurements.

### 2.7. Narrative Review

To support the interpretation of the clinical observations, a narrative review of the literature was conducted. A narrative review approach was selected because the purpose of the literature component was to contextualize and interpret the clinical observations rather than to answer a narrowly defined intervention or diagnostic question requiring systematic evidence synthesis. Relevant literature was identified through searches of PubMed, Scopus, and Google Scholar conducted between March and June 2026. Search terms included combinations of “delirium”, “dementia”, “delirium superimposed on dementia”, “visual impairment”, “sensory deprivation”, “environmental factors”, “psychotic symptoms”, “hallucinations”, “non-pharmacological interventions”, and “pharmacological treatment”. Priority was given to recent peer-reviewed publications, systematic reviews, clinical guidelines, and original studies directly relevant to the clinical phenomena and potential mechanisms discussed in the presented case. Older publications were included when they represented seminal or clinically relevant evidence not adequately replaced by more recent literature. Studies were selected based on their relevance to the interpretation of delirium in dementia, sensory and environmental vulnerability, psychotic manifestations, and management strategies. The literature was synthesized narratively to identify clinically plausible mechanisms and to compare the observed patterns with existing evidence.

## 3. Results

### 3.1. Case Report

A 94-year-old female who has resided in a religious congregation for 60 years, with a medical history significant for Alzheimer’s disease-related dementia, presented with behavioral and psychological symptoms of dementia (BPSD), including marked personality changes, agitation, and episodic confusion. She had previously been treated for delirium occurring in the context of dementia. The patient’s medical history was significant for heart failure, arterial hypertension, hyperthyroidism, and polyarticular osteoarthritis.

On physical examination, vesicular breath sounds were present bilaterally without new abnormal auscultatory findings; a few crackles were noted at the base of the left lung, consistent with previously documented congestive changes. Percussion revealed a normal resonant note. The abdomen was soft and non-tender, with no signs of peritoneal irritation. No dysuric symptoms, pain, or other abnormalities of the urinary tract were observed. No clinical signs of urinary or respiratory tract infection were identified.

Vital signs remained stable: blood pressure 127/68 mmHg, heart rate 90 beats per minute. Arterial oxygen saturation was 94%, improving to 98% following breathing exercises.

In this patient, an approximate fluid balance is being maintained and is slightly positive—within the range of 100–200 mL—as reflected by mild lower leg edema.

Daily fluid intake was estimated at approximately 1.8–2.0 L, with slightly lower urine output. Laboratory investigations show normal levels of sodium, potassium, and creatinine, as well as a satisfactory estimated glomerular filtration rate (eGFR ≈ 67 mL/min/1.73 m^2^), calculated using the MDRD (Modification of Diet in Renal Disease) formula.

She did not report any dysuric symptoms or other typical features of a urinary tract infection (e.g., pain or burning during urination). The color, clarity, and odor of the urine remain within normal limits. The inflammatory marker C-reactive protein (CRP) also remains within the normal range.

Additionally, no other typical signs of infection in other systems were observed. There were no symptoms suggestive of upper respiratory tract infection, and vesicular breath sounds over the lung fields are normal. Chest X-ray (CXR) is normal.

No significant changes were observed on electrocardiography (ECG). Laboratory investigations revealed no clinically relevant abnormalities that could directly explain the patient’s condition (hemoglobin [Hb] 14.1 g/dL, platelet count [PLT] 153 × 10^3^/µL, creatinine 0.81 mg/dL, sodium [Na] 142 mmol/L, potassium [K] 4.09 mmol/L, C-reactive protein [CRP] 0.5 mg/L, thyroid-stimulating hormone [TSH] 3.2 µIU/mL).

Computed tomography (CT) of the head revealed no new or clinically significant changes compared to previous imaging.

The patient’s pharmacotherapy and its potential contribution to behavioral symptoms and delirium are presented in Table 1.

### 3.2. Longitudinal Observation of Clinical Course

The observation period spanned from the diagnosis of Alzheimer’s disease in February 2018 to March 2026. At the initial geriatric assessment, the patient presented with memory impairment, including difficulty recognizing personal belongings and disorientation in time. The Mini-Mental State Examination (MMSE) score was 21/30. Visual impairment was limited to the left eye, and the patient remained fully independent in activities of daily living (ADL score: 6).

Activities of daily living (ADL) include basic self-care functions such as bathing, dressing, eating, toileting, and mobility. The earliest loss of independence involved bathing, marking the onset of a gradual functional decline.

Over the following years, a progressive deterioration in functional status was accompanied by an increasing frequency of delirium episodes. Initially infrequent, these episodes became more common as functional impairment advanced. The course was fluctuating, with periods of relative stability interspersed with exacerbations.

These changes are illustrated in Figure 1. Longitudinal observation indicates a close association between declining ADL performance and increasing delirium burden. Functional capacity decreased in a stepwise manner, ultimately reaching a markedly impaired level. In parallel, delirium episodes evolved from occasional occurrences to episodes several times per week, and at times daily [23].

Symptom fluctuations often appeared in relation to environmental or contextual changes, although this was based on clinical observation rather than formal assessment. A notable shift in the clinical course followed the progression of visual impairment, culminating in severe vision loss after trauma and retinal detachment. This was accompanied by a further increase in delirium frequency and a more pronounced decline in functional status. Clinical deterioration was also observed in temporal association with irregularities in the daily routine. Delirium episodes occurred more frequently on holidays or on days when the usual schedule was disrupted. Visits from relatives who were not part of the patient’s regular environment were likewise associated with a worsening of symptoms.

With advancing visual impairment, the MMSE required modification to account for the patient’s inability to complete visually dependent tasks; consequently, the maximum score was reduced from 30 to 26 points. Despite this adjustment, cognitive decline remained evident, with scores decreasing from 21/30 to 14/26 and eventually 0/26, consistent with advanced dementia.

### 3.3. Clinical Assessment

Progressive functional decline, with the patient becoming dependent on assistance in basic activities of daily living (ADLs) and experiencing recurrent falls, was associated with attempts at independent mobilization.

The patient presented with a disturbance in consciousness of both quantitative and qualitative nature, with marked diurnal fluctuation of symptoms, more pronounced in the afternoon and evening hours. Contact with the patient was impaired—she was easily distractible and had difficulty sustaining attention and engaging in logical conversation. Disorientation in time and place was observed. Attention and cognitive functions were significantly impaired, with difficulties in concentration, following the flow of conversation, and retaining newly acquired information.

The patient’s thought processes were tangential and disorganized. She expressed delusional beliefs involving movement through space and an imminent risk of falling. Vivid, complex visual hallucinations were present, including scenes of flooding and sensations of floating or being transported (e.g., flying in an airplane or traveling in a horse-drawn carriage). These experiences were associated with pronounced anxiety and a persistent sense of falling into an abyss. Autonomic symptoms such as tachycardia and episodes of hyperventilation were observed, along with behavioral manifestations including shouting. Further examples of these symptoms are presented in Table 2.

Severe visual impairment may have contributed to the patient’s perceptual disturbances. The left eye had been blind, with no light perception, for several years, while the right eye showed a markedly restricted visual field due to retinal detachment. In this context, visual hallucinations and disorientation appeared clinically understandable. Visual impairment may also have increased her vulnerability to delirium, particularly by limiting environmental cues and disrupting orientation.

## 4. Discussion

The presented case highlights the complex interplay between advanced Alzheimer’s disease, severe visual impairment, and environmental factors as key contributors to recurrent episodes of delirium. Unlike typical triggers such as infections or metabolic disturbances, the clinical course in this patient appeared to be associated with sensory deprivation and environmental instability of the surrounding environment.

Delirium and dementia often coexist in older adults and influence each other. Delirium has an acute onset, while dementia progresses gradually. Patients with dementia are more vulnerable to delirium, which in turn may accelerate cognitive decline. Preventing delirium is therefore important, as it may help preserve cognitive function [26].

In this case, the main diagnostic challenge was to determine whether the observed symptoms reflected delirium superimposed on Alzheimer’s disease or suggested dementia with Lewy bodies. Evaluation of cognitive domains, including memory, attention, executive and visuospatial functions, as well as verbal fluency, may be helpful, particularly at earlier stages of the disease. It is also worth noting that dementia with Lewy bodies remains underrecognized in everyday clinical practice [27,28,29]. In this patient, the overall clinical picture did not support this diagnosis. There were no clear fluctuations in cognition associated with changes in attention or alertness, no evidence of REM sleep behavior disorder, and no signs of parkinsonism such as bradykinesia, resting tremor, or rigidity at any stage of the disease. Although dementia with Lewy bodies was initially considered due to the presence of visual hallucinations and some variability in cognitive functioning, these features alone were insufficient. The history of a gradually progressive cognitive decline was more consistent with Alzheimer’s disease. Taken together, the findings point to delirium superimposed on Alzheimer’s disease. Recurrent visual hallucinations may raise suspicion of an alternative diagnosis, but in the absence of other core features, they are not decisive [27]. Patients with Alzheimer’s disease are more prone to delirium, which is thought to be related to reduced cognitive reserve. This aligns with the concept of increased brain vulnerability, meaning a lower threshold for developing acute disturbances in response to stressors [26]. Alzheimer’s disease and delirium share several biological mechanisms, including neuroinflammation, cholinergic dysfunction, and oxidative stress. In Alzheimer’s disease, these processes are chronic, whereas delirium represents a transient, acute disruption of the same pathways, often triggered by infection, medication effects, or metabolic imbalance. Recurrent delirium in this context can therefore be understood as a consequence of these overlapping mechanisms rather than evidence of a separate underlying disorder such as dementia with Lewy bodies [30].

Due to visual disturbances—which ultimately progressed to complete bilateral blindness—the patient’s condition was differentiated with Charles Bonnet syndrome taken into account. It is a condition characterized by complex visual hallucinations arising secondary to visual impairment, in the absence of a primary psychiatric disorder, and it most commonly affects older adults [31,32]. In the present case, however, insight into the hallucinatory experiences was absent: the patient perceived the hallucinations as real and did not critically appraise their content. This lack of preserved insight, together with behavioral disturbances in the course of dementia and the established diagnosis of Alzheimer’s disease, strongly argued against Charles Bonnet syndrome.

Delirium should therefore be regarded as an acute condition, particularly in older adults, whose brains are more vulnerable and more susceptible to the cumulative effects of precipitating factors. Identification of both precipitating and predisposing factors allows for the delineation of a plausible pathogenetic model [33]. In the diagnostic process, biological factors should be taken into account. The first of these is dehydration and disturbances in fluid and electrolyte balance. In dehydration, reduced cerebral perfusion occurs, along with electrolyte imbalances (particularly sodium and potassium) and an increase in urea levels, all of which exert a toxic effect on the central nervous system [2,34,35]. In the presented case, measures aimed at preventing delirium were introduced, including regular monitoring of fluid balance. Based on these observations, the patient’s average oral fluid intake was estimated at 1.5–1.8 L per day. Nursing records also indicated that during periods of clinical deterioration, the patient received parenteral hydration in the form of intravenous fluids. Additionally, adequate hydration can be assessed clinically by the condition of the skin and conjunctiva, as well as by laboratory parameters, including appropriate sodium and potassium levels and stable creatinine and eGFR values.

It should be noted that the inflammatory response triggered by infection may directly precipitate delirium. This process is thought to involve pro-inflammatory cytokines (such as IL-6 and TNF-α), activation of microglia, and disruption of the blood–brain barrier, all of which contribute to acute brain dysfunction. The level of inflammatory markers may predict delirium more accurately than the presence of infection itself [36,37].

One possible cause of inflammation in people with dementia is a urinary tract infection (UTI). However, this diagnosis is often an oversimplification. In this group of patients, asymptomatic bacteriuria is frequently present; on its own, it does not require treatment and should not be automatically assumed to be the cause of the symptoms [38,39]. In this patient, the risk of urinary tract infection (UTI) appears relatively low. She does not experience urinary or fecal incontinence and reports her physiological needs appropriately, as confirmed during the Activities of Daily Living (ADL) assessment, although she requires assistance in meeting these needs. For most of the observation period (approximately 5.5 years), the use of incontinence aids was only occasional. The patient also remained adequately hydrated, which supported regular bladder emptying and reduced the risk of urinary retention. The literature emphasizes that the main risk factors for urinary tract infection in older adults are urinary retention and catheterization, whereas urinary incontinence plays a secondary role and often reflects functional impairment rather than being a direct cause of infection. Although urinary tract infection cannot be entirely excluded as a potential trigger of delirium, the available clinical data suggest that it was less likely compared to other possible contributing factors [40,41]. In this case, no evidence of pneumonia, one of the best-established infectious precipitants of delirium in older adults, was identified. Infections such as pneumonia, through inflammatory mechanisms and hypoxia, are known to directly affect brain function and should be considered in the differential diagnosis, even if not confirmed in this patient [42,43].

In the case under analysis, the absence of clear infectious factors, adequate control of fluid and electrolyte balance, and the lack of exacerbations of chronic diseases made common acute infectious, metabolic, or systemic precipitants of delirium less likely. However, the clinical presentation cannot be fully explained as “pure” delirium. In patients with pre-existing cognitive impairment, acute episodes of confusion frequently overlap with the behavioral and psychological symptoms of dementia (BPSD), leading to the emergence of psychotic symptoms and a mixed clinical presentation. Pharmacotherapy represents an important potential confounding factor when interpreting the observed clinical course. The patient received several centrally acting medications, including quetiapine, tiapride, pregabalin, melatonin, and memantine, which could potentially influence alertness, cognition, sleep–wake regulation, and neuropsychiatric symptoms. In this patient, pharmacological interventions were associated with only temporary clinical improvement. The introduction or modification of treatment was at times followed by a reduction in the frequency or duration of delirium episodes and improvement in the sleep–wake rhythm; however, these effects were short-lived. Concomitant treatment with tiapride and quetiapine was also associated with increased drowsiness. With further disease progression, subsequent dose modifications were not accompanied by a clearly observable sustained improvement. Thus, pharmacotherapy cannot be excluded as a contributor to fluctuations in the clinical presentation, but it does not appear to fully account for the recurrent pattern of delirium observed over the eight-year period. Nevertheless, given the retrospective single-patient design, the independent effects of medication, progressive neurocognitive decline, sensory impairment, and environmental circumstances cannot be reliably disentangled.

In this context, current guidelines do not recommend antipsychotic medications as a primary treatment for delirium [6,44,45,46,47,48]. When their use is clinically necessary, they should be administered at the lowest effective dose and for the shortest possible duration [6,49]. Antipsychotic treatment is generally reserved for clinically significant agitation, severe distress, or behaviors that may pose a risk to the patient or others.

Environmental factors in a visually impaired patient with dementia.

The present case is consistent with the vulnerability–stress model proposed by Inouye and Charpentier, as the patient had two major predisposing factors for delirium: dementia and severe visual impairment, the latter representing a form of sensory deprivation. Subsequent studies have repeatedly confirmed that cognitive impairment and sensory deficits are among the strongest risk factors for delirium. Under such conditions, even relatively minor environmental disturbances may contribute to the development of acute neuropsychiatric symptoms in vulnerable individuals [1,3].

Severe visual impairment may have further increased susceptibility to delirium by limiting the sensory cues necessary for maintaining orientation within the environment. In the absence of sufficient external input, the patient may have relied to a greater extent on internally generated perceptual and autobiographical content [1].

In this patient, episodes of delirium appeared to occur more frequently in temporal association with environmental changes than with identifiable episodes of acute physiological deterioration. As shown in Table 3, symptom exacerbations were associated with a range of contextual factors, including changes in caregivers, disruptions of daily routine, periods of immobilization, atypical family events, and time of day. Alterations in mental status were observed particularly during changes in caregiving staff, and interactions with certain individuals were repeatedly associated with changes in perception and the emergence of psychotic symptoms.

In people living with dementia, familiar caregivers may function as external points of reference, and their absence may contribute to increased uncertainty and cognitive disorganization. More recent studies indicate that familiarity constitutes one of the key elements of a supportive environment for people with dementia, and the ability to respond to familiar individuals and stimuli may remain relatively preserved even in advanced stages of the disease [11]. Although changes in caregivers are not classically recognized as direct precipitants of delirium, our observations suggest that, in individuals with advanced dementia and severe visual impairment, disruption of relational continuity may represent a significant environmental stressor [50,51]. Recent reviews suggest that the presence of family members and familiar caregivers may contribute to a greater sense of security, improved orientation, and reduced distress associated with delirium [7].

Given the temporal association between symptom exacerbations and environmental instability observed in this patient, non-pharmacological interventions were designed to address the contextual factors identified in Table 3. A person-centred care (PCC) approach was adopted to improve environmental predictability, preserve continuity of care, and reduce sensory and emotional stressors.

The present case raises the possibility that environmental instability may represent an important contributing factor in the development of delirium when neurodegenerative changes and sensory deprivation substantially limit the patient’s adaptive capacity [52,53].

### 4.1. Clinical Implications and Environmental Modulation

Because symptom exacerbations were frequently observed in temporal association with environmental instability and sensory disorientation, interventions were primarily aimed at increasing environmental predictability, maintaining continuity of care, and reducing anxiety-provoking stimuli. Accordingly, a person-centred approach was adopted, with therapeutic strategies tailored to the patient’s sensory limitations, previous routines, and preserved emotional responses. These observations should be interpreted as hypothesis-generating and require confirmation in prospective studies involving larger patient populations.

### 4.2. Pharmacological Considerations

Pharmacological management was complicated by the coexistence of advanced dementia, recurrent psychotic symptoms, severe visual impairment, and a pronounced anxiety component. Current guidelines emphasize that antipsychotic medications do not treat the underlying cause of delirium and should be used primarily in cases of significant agitation, patient distress, or safety concerns [10]. Quetiapine constituted the main component of treatment; however, doses exceeding 100 mg were associated with excessive sedation, rigidity, and an increased risk of falls. These adverse effects are consistent with mechanisms described in the literature regarding the increased risk of falls associated with antipsychotic therapy in older adults with cognitive impairment [54]. Combination therapy with quetiapine and tiapride increased drowsiness but also appeared to be clinically associated with shorter delirium episodes. It is possible that this effect was partly mediated by improved organization of the sleep–wake cycle, disturbances of which are considered an important component of delirium pathophysiology [54,55,56].

Melatonin represented a useful adjunctive intervention, contributing to improved regulation of circadian rhythms. Although its efficacy in the treatment of delirium has not been conclusively established, accumulating evidence suggests that interventions aimed at normalizing circadian rhythms may provide clinical benefits [19,57,58]. Pregabalin proved to be a particularly challenging therapeutic consideration. Despite concerns regarding its potential adverse effects on cognitive function and its possible association with delirium in older adults, its use was associated with partial reduction in anxiety symptoms and improved behavioral control. The observed effect appeared to result primarily from alleviation of anxiety and emotional distress rather than from a direct effect on delirium itself.

Attempts to achieve symptom control with antidepressant and anxiolytic therapies, including mirtazapine, serotonergic agents, and alternative antipsychotics such as olanzapine, did not result in sustained clinical improvement. Initial improvements in anxiety and agitation were transient and were subsequently followed by symptom recurrence despite dose escalation. These observations may suggest limited benefit from further intensification of psychotropic treatment when the clinical course is largely determined by advanced neurodegeneration, sensory deprivation, and environmental factors. However, this hypothesis requires confirmation in further studies [59].

Overall, pharmacological treatment alone did not provide stable symptom control and appeared to play a supportive rather than central role in the therapeutic process. The literature emphasizes that the use of antipsychotic medications in older adults requires continuous assessment of the balance between benefits and risks, as well as individualized treatment decisions [54]. In this patient, medications likely modified the threshold for neuropsychiatric decompensation but did not address the environmental and sensory factors that appeared to sustain recurrent episodes of delirium [60].

### 4.3. Non-Pharmacological Interventions

Our observations are consistent with current guidelines, which recognize multicomponent non-pharmacological interventions as the cornerstone of delirium prevention and supportive management, while pharmacological treatment should be reserved as an adjunctive approach. Particular emphasis is placed on optimizing the patient’s physical condition, maintaining adequate hydration, monitoring for infections, and ensuring appropriate orientation and environmental stability [6,61]. According to the latest Cochrane systematic review, multicomponent non-pharmacological interventions reduce the risk of delirium by approximately 43% and may shorten both hospital length of stay and the duration of delirium episodes, although their effect on delirium severity remains uncertain [62].

The structured involvement of family members in daily care is consistent with current recommendations emphasizing family engagement as an important component of multicomponent non-pharmacological delirium management. Previous studies, particularly among older patients undergoing orthopedic surgery, have shown that educating and actively involving informal caregivers can improve patient orientation, reduce anxiety, and optimize the quality of care, although implementing family-integrated care may present organizational and educational challenges [63,64]. In the present case, limiting visits to a single family member, providing caregiver education, and involving relatives in structured daily activities and supported reminiscence therapy appeared to reduce emotional distress and improve acceptance of the patient’s fluctuating condition. These observations suggest that family-centered interventions may also be beneficial in patients with delirium superimposed on dementia and severe visual impairment [12].

Maintaining a structured daily routine and adapting everyday activities to the patient’s previous lifestyle are consistent with current person-centred dementia care recommendations, which emphasize the importance of familiarity and predictability in reducing behavioral symptoms and supporting orientation [65]. In the present case, familiar household activities appeared to provide meaningful engagement without causing excessive cognitive burden. In contrast, bathing was repeatedly temporally associated with the emergence of delusions centered on physically demanding work. This observation suggests that personal care activities may activate autobiographical memories and previously learned behavioral schemas, particularly when they are perceived as physically demanding or emotionally stressful. Modifying the bathing procedure by reducing physical effort, preserving familiar sensory cues, and encouraging the patient’s active participation appeared to reduce distress and improve cooperation [66,67]. These findings support the concept that seemingly routine care activities should be individually adapted, as their subjective meaning may substantially influence behavioral and neuropsychiatric symptoms in patients with advanced dementia and delirium. We hypothesize that bathing may have served as a context associated with autobiographical memory activation rather than merely representing a stressful care activity. In the setting of advanced dementia and delirium, impaired reality monitoring may have contributed to these activated memories being experienced as ongoing events, thereby contributing to the development of work-related delusions. This interpretation is consistent with person-centered dementia care, which recognizes that the subjective meaning of everyday activities may substantially influence behavioral and psychological symptoms [68].

Music-based interventions, particularly those involving familiar songs, have consistently been associated with reductions in agitation and anxiety in people with dementia [69,70,71]. In the present case, singing religious songs may have additionally promoted emotional security through highly familiar autobiographical and spiritual cues. In the case presented here, spirituality and religious practices played a special role. Although evidence regarding structured religious interventions in delirium remains scarce, a growing body of literature highlights the importance of spirituality in dementia care. Studies suggest that spiritual well-being is associated with a lower severity of behavioral and psychological symptoms of dementia, slower cognitive decline, and improved psychological well-being. Spiritual needs persist throughout all stages of dementia, with prayer consistently identified as one of the most frequently expressed needs [72,73,74,75]. Religious practices may help individuals preserve their sense of identity, maintain social connectedness, cope with illness, reduce psychological distress, and foster hope and meaning despite progressive cognitive decline. Consequently, current recommendations advocate for the individualization of spiritual care according to the patient’s previous beliefs, values, and lifelong religious practices [76]. In the present case, the repetitive rhythm of prayer appeared to function as a familiar cognitive and emotional anchor, promoting calmness and behavioural stability. We hypothesize that this effect was related not only to the spiritual significance of prayer but also to its highly familiar, predictable, and repetitive nature, which may have enhanced orientation and reduced anxiety. This observation should be interpreted as an individual, context-dependent response rather than evidence of a specific therapeutic effect of prayer on delirium.

Despite targeting different aspects of care, the non-pharmacological interventions that were associated with clinical improvement appeared to share a common feature: reducing environmental uncertainty and supporting orientation through familiarity, predictability, and the preservation of personally meaningful routines. In patients with delirium superimposed on dementia, impaired attention and reality monitoring markedly reduce the ability to interpret changing environmental stimuli. Consequently, interventions that minimized uncertainty and reinforced consistent cognitive and emotional cues may have lowered the burden on already compromised attentional networks, thereby reducing the likelihood of hallucinations, delusions, and fluctuating confusion.

## 5. Conclusions

Delirium superimposed on dementia is a complex neuropsychiatric syndrome requiring an individualized, person-centered approach. Recurrent delirium episodes may substantially worsen cognitive function, functional independence, and overall quality of life in patients with dementia.

In addition to established biological precipitants, disruption of environmental continuity may contribute to the development of delirium in particularly vulnerable individuals. In the presented case, progressive visual impairment appeared to increase susceptibility to environmental changes, suggesting that sensory deprivation may amplify the effects of contextual stressors.

In this patient, individualized non-pharmacological interventions tailored to the patient’s sensory limitations, previous lifestyle, and emotional needs were associated with a reduction in the frequency of delirium episodes and psychotic symptoms, whereas pharmacological treatment alone did not provide sustained clinical stabilization.

Interventions aimed at strengthening the patient’s sense of security and preserving familiar environmental cues appeared to be the most beneficial. Familiar routines, continuity of care, and personally meaningful activities, including prayer and other long-established religious practices, were associated with improved behavioral stability and reduced emotional distress.

These findings suggest a potential association between progressive visual deprivation and increased delirium frequency, functional decline, and changes in cognitive performance, highlighting areas for further investigation. Further studies are needed to clarify the role of sensory deprivation and environmental continuity in the pathophysiology and prevention of delirium.

The proposed interaction between sensory deprivation, environmental instability, and recurrent delirium remains hypothesis-generating and requires confirmation in prospective studies involving larger patient populations.

### Limitations

This study has several limitations. First, it is based on a single-patient case, which limits the generalizability of the findings. Consequently, the observed associations between progressive visual impairment, environmental continuity, and recurrent delirium should be interpreted as hypothesis-generating rather than causal.

Second, although the patient was observed longitudinally over an eight-year period, the frequency and characteristics of delirium episodes were partly derived from routine nursing and geriatric documentation and clinical observations rather than from prospectively standardized assessments performed at predefined time points, which is mentioned in the Methods part of the manuscript.

Third, the coexistence of advanced Alzheimer’s disease, progressive visual impairment, behavioral and psychological symptoms of dementia, and pharmacological treatment makes it difficult to determine the independent contribution of individual factors to the clinical course.

Finally, as this study combines a longitudinal case analysis with a narrative review, the conclusions are intended to provide clinical insights and generate hypotheses rather than establish causal relationships. Future prospective studies involving larger patient populations are needed to clarify the role of sensory deprivation and environmental continuity in the development and prevention of delirium in dementia.

In addition, environmental factors and anxiety-related responses were assessed through longitudinal clinical observations rather than validated environmental or psychological assessment instruments.

## Figures and Tables

**Table 1:** Chronic medications and their potential contribution to delirium and behavioral and psychological symptoms of dementia [19,20,21,22].

Medication	Indication	Dose	Anticholinergic Burden	Delirium Risk	Potential Impact on BPSD	Comments
Bisoprolol	Heart failure, arrhythmia	1.25 mg/day	Low	Low–Moderate	Possible (fatigue, depressive symptoms)	β-blockers may rarely contribute to fatigue, sleep disturbance, or depressive symptoms
Methimazole (Metizol)	Thyrotoxicosis	5 mg every other day	None	Low	Unlikely	Thyroid dysfunction itself may influence cognition more than treatment
Potassium (Kalipoz)	Electrolyte supplementation	391 mg/day	None	Low	Unlikely	No direct CNS effects unless electrolyte imbalance occurs
Memantine (Polmatine)	Alzheimer’s disease	20 mg/day	Low	Low	Possibly beneficial	May reduce behavioral symptoms in dementia
Quetiapine (Kwetaplex SR)	Behavioral symptoms	50–100 mg/day	Low	Low–Moderate	Beneficial/Sedative	Used for BPSD; may cause sedation and increase fall risk
Pregabalin	Neuropathic pain/anxiety	150 mg/day	Low	Moderate	Remains equivocal	Associated with dizziness, somnolence, and delirium risk in elderly
Tiapride	Behavioral disturbances	100 mg/day	Low	Moderate	Mixed (beneficial vs. sedation)	Dopaminergic blockade; may cause sedation and extrapyramidal symptoms
Melatonin	Sleep disturbances	5 mg/night	None	Low	Beneficial	May help regulate sleep–wake cycle and reduce delirium risk

**Table 2:** Examples of Patient’s Verbalizations and Their Psychopathological Interpretation [5,24,25].

Patient’s Words	Primary Symptom	Secondary Features	Psychopathological Interpretation
*Please do not touch me because I’m about to fall out of this airplane (the patient has never traveled by airplane).*	Delusion (misinterpretation)	Anxiety, visual imagery	Delusional misinterpretation based on impaired perception and spatial disorientation
*This cart is about to fall off a cliff.*	Visual hallucination	Anxiety	Complex, dynamic visual hallucination with threat content
*The hay wagon is tipping over and is about to crush me.*	Visual hallucination	Fear	Formed visual hallucination with catastrophic interpretation
*There is a henhouse over a precipice; I am inside it and we are about to fall.*	Visual hallucination and delusion	Spatial disorientation	Immersive hallucination with loss of environmental boundaries
*Oh, here stands a little cow and a little horse, and snow is falling on them.*	Visual hallucination	Neutral affect	Non-threatening, formed visual hallucination
*Who has ever seen anyone eat potatoes and noodles with poppy seeds? A disgrace, a disgrace of the assembly (she said while eating tomato soup).*	Disorganized thinking	Incongruent affect	Context-inappropriate, tangential speech
*I’m going to work, please don’t stop me, I have a lot of dishes to wash here (she said while in bed in the middle of the night).*	Delusion (temporal disorientation)	Confusion	Intrusion of past roles due to impaired orientation
*How long will we be standing here, because the rain is falling on me (said while sitting in an armchair in the late afternoon).*	Delusion (misinterpretation)	Anxiety, disorientation	Misinterpretation of environmental context with impaired orientation and possible tactile/visual hallucination

**Table 3:** Contextual Factors and Symptom Exacerbation.

Contextual Factor	Observed Symptoms	Possible Explanatory Model
Change of caregiver/absence of primary caregiver	Anxiety, agitation	Loss of familiarity and reduced sense of security
Disruption of daily routine	Visual hallucinations, confusion	Impaired temporal orientation and cognitive instability
Holidays or atypical days	Psychotic symptoms (hallucinations, delusions)	Environmental unpredictability and overstimulation
Immobilization/reduced activity	Intrusions of past experiences	Reduced external input with possible increased reliance on internally generated content
Late afternoon (evening hours)	Hallucinations, anxiety	Sundowning effect with worsening attention and perception
Family visits (especially distant relatives)	Hallucinations, increased confusion	Emotional overstimulation and unfamiliar stimuli
Severe visual impairment	Visual hallucinations	Sensory deprivation and impaired visual processing
Anxiety-related internal imagery	Recurrent themes of falling, threat, catastrophe	Threat bias with impaired reality monitoring
Bathing	Intrusions of work-related or physically demanding activities	Hypothetical activation of autobiographical schemas associated with fatigue and reduced cognitive control

## Data Availability

The clinical data underlying this case report are not publicly available due to patient privacy and confidentiality considerations. Relevant anonymized data are included in the article.
